# Sex-specific insights into drug-induced lifespan extension and weight loss in mice

**DOI:** 10.1038/s41514-025-00229-w

**Published:** 2025-05-19

**Authors:** Aleksey V. Belikov, Angelo Talay, João Pedro de Magalhães

**Affiliations:** Genomics of Ageing and Rejuvenation Lab, Department of Inflammation and Ageing, School of Infection, Inflammation and Immunology, College of Medicine and Health, University of Birmingham, Birmingham, UK

**Keywords:** Chemical biology, Ageing

## Abstract

The DrugAge database serves as a comprehensive resource for studying compounds that increase lifespan in model organisms. In the latest version of DrugAge, we implemented multiple updates, predominantly focusing on mouse studies to enhance data accuracy and consistency. Key improvements include re-recording of mouse data from original sources, standardization of drug dosages to parts per million, and recording of administration routes, treatment initiation ages, and lifespans of controls. The user interface was also upgraded. Additionally, weight change data were included to address the potential impact of caloric restriction induced by drug administration on lifespan. Our analysis revealed significant correlations between weight loss and lifespan extension in male mice, particularly in studies conducted by the Interventions Testing Program, highlighting the importance of considering weight changes in lifespan studies. We also observed notable sex-related differences in lifespan and weight change responses, underscoring the need for sex-specific analyses in aging research.

## Introduction

DrugAge (https://genomics.senescence.info/drugs/) is a comprehensive database that compiles a wide range of approved and investigational drugs, chemical compounds, and plant extracts, collectively referred to as “compounds,” which have shown effects on the lifespan of healthy model organisms such as mice (*Mus musculus*), flies (*Drosophila melanogaster*), and worms (*Caenorhabditis elegans*)^1,2^. The database records both significant and non-significant extensions of mean, median and maximal lifespan. Maintained with rigorous standards, DrugAge ensures all entries are manually curated from the scientific literature. The focus is on compounds potentially impacting aging, and we excluded those tested on disease-prone animals or under harmful conditions. Most negative results are excluded unless particularly relevant to the field of aging research. Each entry corresponds to a specific observation from a study, allowing for multiple, potentially conflicting entries for a single compound, thus encouraging users to interpret the data independently.

The DrugAge homepage facilitates direct searches for specific compounds, species, or bibliography references and allows database downloads. The Browse page displays all entries, which can be filtered or sorted by criteria such as compound, species, strain, dosage, sex, and lifespan changes. PubMed references are provided for further exploration. Individual compound and species pages offer a summary table listing lifespan changes across experiments, and a results table showing detailed data. DrugAge provides visual data representation through lifespan charts, which can toggle between average and maximal lifespan changes, displaying significant and non-significant results or distinguishing male versus female results. The Drug Data Summary page features pie charts illustrating compound and species representations across studies, while the Statistics page offers a summary of assays, compounds, species, references, and the maximum lifespan changes observed for each species. DrugAge Build 5 features over 1000 compounds evaluated in over 3400 experiments across 35 species, supported by more than 660 references.

One of the major updates to Build 5 was the inclusion of weight change data for murine studies. Caloric restriction is the most reproducible longevity intervention, demonstrating the magnitude of the effect on lifespan higher than any compound tested. Caloric restriction may work by preventing obesity^3^, by retarding growth and development if started early in life^4^ or by other mechanisms^5^. In fact, the magnitude of lifespan extension under caloric restriction has r = 0.99 correlation with weight gain in control mice across various strains^6^. Compounds may change the smell, taste or appearance of chow to which they are usually added in murine experiments, leading to reduced food consumption and inadvertent caloric restriction. Moreover, some compounds can affect the appetite or feeding behaviour by interfering with dopamine reward systems, leptin levels or circadian rhythms. In fact, it has been proposed that many of the well-known longevity drugs are caloric restriction mimetics^3,7^. Thus, controlling for weight change while testing the effects of various compounds on lifespan is crucial. Here, we demonstrate that pharmacological lifespan extension significantly correlates with weight loss in male mice, particularly in studies conducted by the Interventions Testing Program (ITP)^8,9^.

## Results

### Build 5 updates

For build 5 of DrugAge we implemented multiple updates and improvements. Most of the changes concerned data from murine studies (https://genomics.senescence.info/drugs/species_details.php?species_name=Mus+musculus). All mouse data was re-recorded from source papers, both for previously included and new studies, to increase accuracy and consistency. Drug dosages were converted to ppm (parts per million, 1 ppm = 1 mg/kg, or 1 μg/g, or 1 mg/l, or 1 μg/ml, or 0.0001% solution) as per ITP standard^9^. Administration route and dosing description were standardised and clearly indicated as “food”, ”water”, ”bodyweight”, ”injection” or ”gavage”, reflecting four major types of drug administration and dosing in rodents – ppm of chow, ppm of drinking water, ppm of bodyweight injection and ppm of bodyweight gavage. Age at initiation and treatment duration were recorded from source papers. Separate statistical significance was recorded for average/median and maximum lifespan extension, where available. Average/median control lifespan was recorded to facilitate interpretation of results amidst concerns about short-lived controls^10^. Weight change was calculated from tables or approximated from plots where data tables or other exact values were not available. Typically, the time point with the maximal weight difference between the drug and the control was chosen. If that was not clear from the publication, then the median survival time point was selected. Weight change statistical significance was also recorded where available. ITP studies were clearly marked, as the ITP is considered a gold standard in murine lifespan studies due to the use of three independent experimental sites and large cohorts of genetically heterogeneous mice^8,9^.

In the whole database, including other species, some compounds’ duplicates with different names but the same PubChem ID were merged. This explains slight reduction in the number of drugs compared to the previous build despite adding new experiments. Plant and other extracts were uniformly named to contain “extract” in their name, to avoid duplicates and confusion with isolated chemicals. Sex and significance data were converted from string format to multiple choice (dropdown menu) format to remove duplicates and facilitate data selection.

Regarding the user interface, we added a “Sex” column and an option to indicate male and female data on the graph with distinct colours. Sex differences in lifespan effects are often dramatic so these additions were crucial. Please note that other species beyond mice might not always have sex data. “MEAN(Avg Lifespan Change %)” and “MEAN(Max Lifespan Change %)” columns were added to the Summary tables, in addition to the previously available MAX option. This allows users to rank drugs more confidently without the risk of relying on outliers. We made clicking on a PMID in the last column to display all entries related to a given study. This facilitates comparisons between sexes, as well as between different dosages, treatment durations or even mouse strains, if tested within the same study. We made graphs to display the mean and standard deviation of lifespan changes for each compound, combining individual experiments together, which greatly reduced the length and complexity of the graphs. It is also now possible to select only significant results to be displayed on the graph. Additionally, we made graphs to always display zero on the “Lifespan Change (%)” axis to more consistently reflect the relative magnitude of effects, especially in cases where all drugs are very effective, and the poorly performing comparators are missing. Numerous graph and table display issues were also fixed. For *Mus musculus* page specifically, we added “Age at initiation” (search), “Treatment duration” (search), “Max Lifespan Significance” (dropdown), “Avg/Med Control Lifespan (days)” (slider), “Weight Change (%)” (slider), “Weight Change Significance” (dropdown) and “ITP” (dropdown) columns to display the expanded data recorded from source articles.

### Murine studies statistics

DrugAge Build 5 *Mus musculus* section features 134 compounds evaluated in 373 experiments, where one experiment refers to a particular dosage and treatment scheme of a compound tested in a particular sex in a particular strain. DrugAge lists 198 experiments for male mice and 172 for females, and males also have a higher number of experiments with significant average/median lifespan extension – 72 (36%) vs 50 (29%) for females. Top 10 experiments in *Mus musculus* ranked by significant average/median lifespan extension are listed in Table 1 for males and Table 2 for females. As can be seen, most suscessful treatments were initiated within the first 4 months of life, with the exception of rapamycin, X203 (anti-IL-11) and 17-alpha-estradiol, which were initiated at 9 months or later. Most compounds were delivered in chow, as this is the easiest option. Many compounds increasing average/median lifespan also extend maximum lifespan. Most of the top experiments are not from the ITP, with independent validation pending. Moreover, several of them are from 1970s, 1980s, or even 1960s and 1950s, and require reproduction in modern labs. Many compounds induced weight loss, which will be discussed in the next section.

**Table 1:** Top 10 experiments in *Mus musculus* ranked by significant average/median lifespan extension in males

Compound	Strain	Dosage	Age at initiation	Avg/med lifespan change	Max lifespan change	Weight change	ITP	PMID	Year
Butylated hydroxytoluene	BaLB/c	7500 ppm food	2.6 months	30%	NS	NA	No	448040	1979
Levodopa	Swiss Albino	40,000 ppm food	1 month	28%	12%	−13%	No	850799	1977
Royal Jelly	C3H/HeJ	50 ppm food	2 months	26%	NS	NA	No	12954483	2003
Royal Jelly	C3H/HeJ	500 ppm food	2 months	24%	NS	NA	No	12954483	2003
Spermidine	C57BL/6	3 mM water	after weaning	24%	NA	NS	No	28386016	2017
Rapamycin	UM-HET3	42 ppm food	9 months	23%	8%	−19%	Yes	24341993	2014
Acarbose	UM-HET3	1000 ppm food	4 months	22%	11%	−15%	Yes	24245565	2014
X203 (anti-IL-11)	C57BL/6 J	40 ppm bodyweight monthly i/p injection	17.2 months	22%	NA	NA	No	39020175	2024
Butylated hydroxytoluene	BaLB/c	7500 ppm food	2 months	22%	NS	NA	No	448040	1979
17-alpha-estradiol	UM-HET3	14.4 ppm food	10 months	19%	12%	−15%	Yes	27312235	2016

*NS* not significant, *NA* not available.

**Table 2:** Top 10 experiments in *Mus musculus* ranked by significant average/median lifespan extension in females

Compound	Strain	Dosage	Age at initiation	Avg/med lifespan change	Max lifespan change	Weight change	ITP	PMID	Year
Metformin	SHR	100 ppm water	3 months	38%	21%	NS	No	18728386	2008
Pineal gland extract	C3H/Sn	0.5 mg subcutaneous injection 5 days monthly	3.5 months	31%	14%	NA	No	6752596	1982
Thymus extract	C3H/Sn	0.5 mg subcutaneous injection 5 days monthly	3.5 months	28%	11%	NA	No	6752596	1982
2-mercaptoethylamine hydrochloride	C3H	10,000 ppm food	after weaning	26%	NA	−1%	No	13711616	1961
Rapamycin	UM-HET3	42 ppm food	9 months	26%	11%	−20%	Yes	24341993	2014
X203 (anti-IL-11)	C57BL/6 J	40 ppm bodyweight monthly intraperitoneal injection	17.2 months	25%	NA	NA	No	39020175	2024
Butylated hydroxytoluene	BaLB/c	7500 ppm food	2.6 months	25%	NS	NA	No	448040	1979
Rapamycin	UM-HET3	14 ppm food	9 months	21%	11%	−4%	Yes	24341993	2014
Phenformin	C3H/Sn	2 mg oral gavage 5 times a week	3.5 months	21%	28%	NA	No	14618027	2003
Calcium pantothenate	C57BL/6	300 µg/day in water	1 month	20%	NA	12%	No	13614445	1958

*NS* not significant, *NA* not available.

**Fig. 1:**
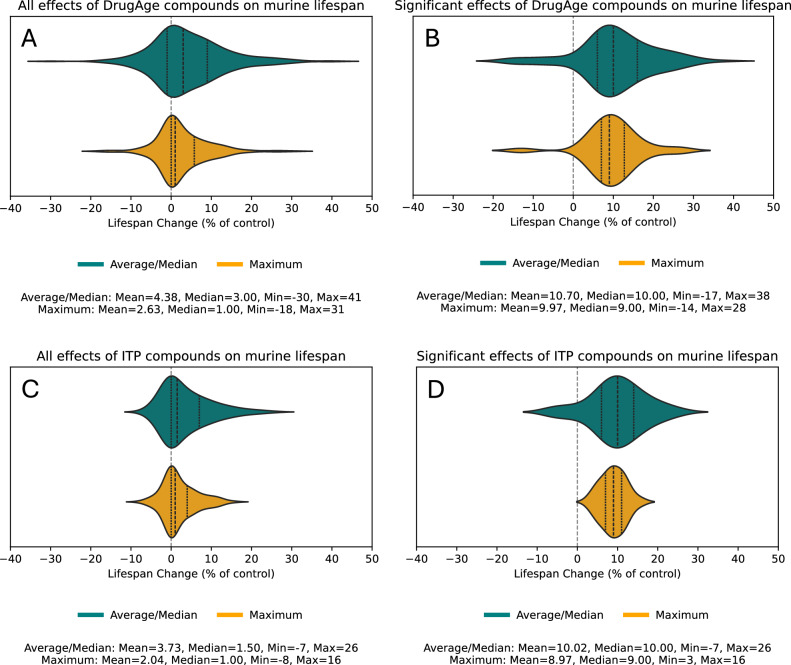
Effects of compounds on average/median and maximum murine lifespan. **A**, **B** Compounds from DrugAge. **C**, **D** Compounds from ITP studies. **A**, **C** All effects. **B**, **D** Significant effects. The significance of the effects was taken from the source publications. See Supplementary Fig. 1 for sex-specific plots.

Effects of DrugAge-listed compounds on the average/median murine lifespan range from −30 to +41 percent of control, resulting in 4.4% extension on average (Fig. 1A). Effects on the maximum lifespan range from −18 to +31 percent of control, resulting in 2.6% extension on average (Fig. 1A). When only statistically significant effects on the average/median murine lifespan are considered, they range from −17 to +38 percent of control, resulting in 10.7% extension on average (Fig. 1B). Significant effects on the maximum lifespan range from −14 to +28 percent of control, resulting in 10% extension on average (Fig. 1B). This might reflect the size of the effect (around 10%) required to achieve statistical significance in a typical murine lifespan experiment.

When only the ITP studies are considered, effects on the average/median murine lifespan range from −7 to +26 percent of control, resulting in 3.7% extension on average (Fig. 1C). Interestingly, this extension is much more pronounced in males (5.5%) than in females (1.6%) (Supplementary Fig 1C). Effects on the maximum lifespan in ITP studies range from −8 to +16 percent of control, resulting in a 2% extension on average (Fig. 1C). Less negative effects on the lifespan in ITP studies are likely explained by careful selection of compounds for testing, usually based on previous successful studies^8,9^. Finally, when only statistically significant effects on the average/median murine lifespan in the ITP studies are considered, they range from −7 to +26 percent of control, resulting in 10% extension on average (Fig. 1D). This extension is also more pronounced in males (11.8%) than in females (6.7%) (Supplementary Fig 1D). Significant effects on the maximum lifespan in ITP studies range from +3 to +16 percent of control, resulting in a 9% extension on average (Fig. 1D).

The average/median lifespan change positively correlates with the maximum lifespan change, both in males and females, with females consistently achieving higher and more significant correlation (Supplementary Fig. 2). The correlation is very strong in the full ITP dataset, both in males (slope: 0.46, R2: 0.47, *p*-value: 7 × 10^−13^) and females (slope: 0.58, R^2^: 0.57, *p*-value: 5 × 10^−16^)(Supplementary Fig 2C). This might be due to a consistent way of measuring both median (50% survival) and maximum (10% survival) lifespan in ITP studies^8,9^.

### Weight change analysis

**Fig. 2:**
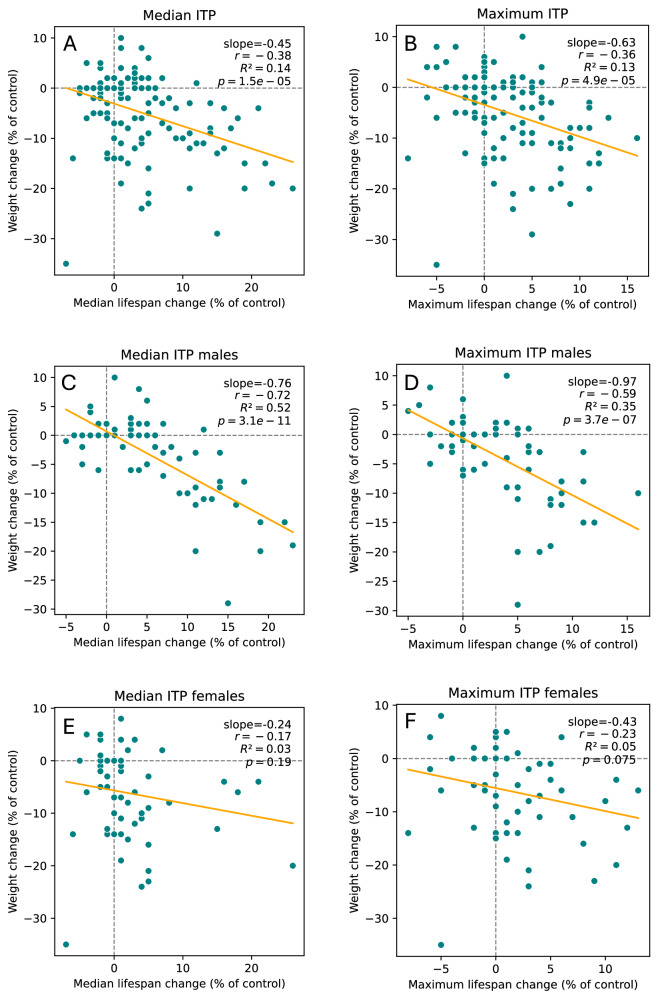
Correlations between weight change and lifespan change for compounds from ITP studies. **A**, **C**, **E** Median lifespan change. **B**, **D**, **F** Maximum lifespan change. **A**, **B** Both sexes combined. **C**, **D** Males. **E**, **F** Females. The linear regression and the corresponding statistics were produced using the linregress function from the scipy.stats library in Python.

When all data are considered, correlations between weight change and median or maximum lifespan change are significant for males and females combined and for males alone but not for females alone (Supplementary Fig 3). Nevertheless, the R^2^ and slopes are small, even for males. However, when ITP-only data are used, *p*-values, R^2^ and slopes are much larger (Fig. 2). In males there is a strong correlation between the increase in lifespan and decrease in weight, both for median (slope: −0.76, R^2^: 0.52, *p*-value: 3.09 × 10^−11^, Fig. 2C) and for maximum (slope: −0.97, R^2^: 0.35, *p*-value: 3.73 × 10^−7^, Fig. 2D) lifespan. Curiously, in females many compounds led to dramatic decrease in weight without substantial increase in lifespan (Fig. 2E). Correlations might be more pronounced in ITP studies due to higher quality and uniformity of the data, being collected for all drugs at the same sites, in the same conditions, using the same protocols (incl. for weight measurements) and using large cohorts of genetically heterogeneous mice^8,9^.

**Fig. 3:**
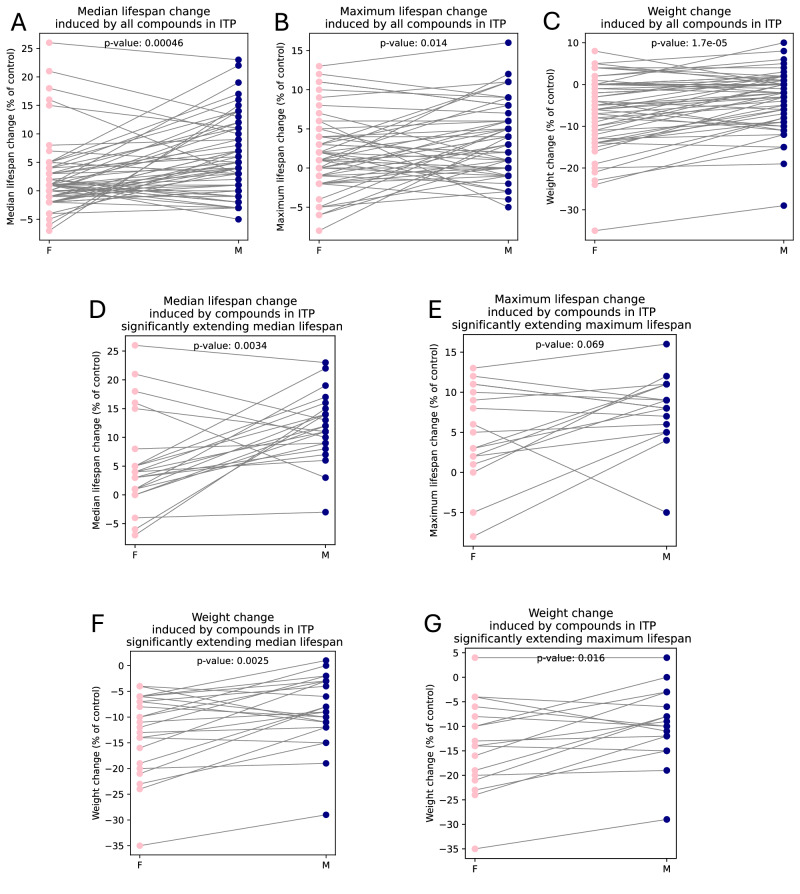
Pairwise comparisons between males (M, navy) and females (F, pink) for compounds from ITP studies. **A**, **D** Median lifespan change. **B**, **E** Maximum lifespan change. **C**, **F**, **G** Weight change. **A**, **B**, **C** All tested compounds were considered. **D**, **F** Only compounds significantly extending median lifespan were considered. **E**, **G** Only compounds significantly extending maximum lifespan were considered. Lines represent individual compounds. The significance of the lifespan effects was taken from the source publications. The significance of the differences between males and females was estimated by a paired *t*-test using the ttest_rel function from the scipy.stats library in Python.

Because we observed larger correlations in males than in females, we decided to look at sex-related differences more closely. We performed pairwise comparisons for each drug that has been tested in both males and females by ITP. When all such compounds are considered, including the ones not extending lifespan significantly, the difference between males and females in median lifespan change (*p*-value: 4.58 × 10^−4^, Fig. 3A) and weight change (*p*-value: 1.66 × 10^−5^, Fig. 3C) is more significant than in maximum lifespan change (p-value: 0.0144, Fig. 3B). For the majority of the compounds, males have higher median lifespan extension than females but less pronounced weight loss, but there are exceptions. Plotting pairwise lifespan and weight changes on the same graph confirms that the same compound can typically induce stronger weight loss in females but stronger median lifespan extension in males (Supplementary Fig. 4). The results for maximum lifespan extension are less clear.

When focusing specifically on compounds significantly extending median lifespan in ITP studies (in at least one sex), the higher median lifespan increase (Fig. 3D) and less pronounced weight loss (Fig. 3F) in males compared to females remained statistically significant. Curiously, while there was no significant difference between males and females in the magnitude of maximum lifespan increase amongst compounds that significantly extended maximum lifespan in ITP studies (Fig. 3E), females still had significantly more pronounced weight loss than males when receiving those compounds (Fig. 3G).

## Discussion

Our study examined the relationship between weight change and lifespan extension in mice treated with various compounds, revealing significant sex-specific differences. The results indicate that weight loss correlates strongly with increased lifespan in males but not in females, suggesting that the mechanisms by which drugs affect longevity may differ between sexes.

Several studies in genetically heterogeneous mice (the same kind as used in ITP studies) highlighted a different relationship between bodyweight and lifespan in males compared to females. One study showed that the difference in survival between heavy and light mice is much more pronounced in males than in females, with heavy males on average living less than heavy females, and light males living longer than light females^11^. Another study showed that lifespan negatively correlates with weight in males, especially at younger ages, but there is almost no correlation in females^12^. Notably, that study also clearly demonstrated that survival in the first two-thirds of lifespan is much worse for males compared to females^12^. Similarly, in a recent study, long-lived males were lighter than short-lived males, whereas there was almost no difference in weight between long-lived and short-lived females^13^. Consistent with this, in humans, being overweight in adolescence is associated with an increased risk of mortality from all causes, coronary heart disease, atherosclerotic cerebrovascular disease, and colorectal cancer among men, but not among women^14^.

Interestingly, male mice have a locus on chromosome 9 which may be modulating longevity through its effect on growth or body weight, but no such locus has been found in females^13^. Top-scoring genes in this locus are *Glb1*, which is a beta-galactosidase, and *Rtp3*, which is a receptor-transporting protein that promotes functional cell surface expression of the bitter taste receptors TAS2R16 and TAS2R43. We speculate that higher expression of Rtp3 in some males might make them consume less chow due to more pronounced bitterness and thus reduce weight and promote longevity.

Compounds that decrease weight in animals may function as caloric restriction mimetics^3,7^. The pronounced weight loss without a corresponding increase in lifespan we noted in female mice treated with various compounds suggests that weight reduction is not the main mechanism of lifespan extension for this sex. In the seminal 1935 experiment in rats by McCay, Crowell and Maynard, caloric restriction retarded growth of both males and females but extended lifespan only in males^4^. One alternative to caloric restriction aimed to achieve similar lifespan extension effects is protein restriction, such as restriction of methionine or branched chain amino acids leucine, isoleucine and valine^7^. Lifelong restriction of branched chain amino acids reduced weight in C57BL/6 J females more than in males, but led to a 30% increase in lifespan and a reduction in frailty only in males^15^. This diet also affected mTOR and FoxO pathways only in males^15^.

This divergence between sexes in lifespan responses to compounds, dietary restriction and weight loss may be due to different metabolic, hormonal, developmental or genetic profiles. For instance, females might experience more pronounced metabolic adaptations or hormonal fluctuations in response to caloric restriction that do not necessarily translate to increased longevity. In fact, it has been recently shown in a study of female genetically heterogeneous mice that the top *within-diet* predictor of long lifespan was the ability of mice to *retain* bodyweight under caloric restriction, an indicator of stress resilience^16^. Even more surprisingly, in female mice, lean tissue mass was negatively correlated with lifespan, more strongly early in life, while adiposity was positively correlated with lifespan, more prominently in late life^16^. Moreover, fasting glucose and energy expenditure were not associated with lifespan^16^. This evidence argues against the reduction in obesity or in metabolism as the mechanism for lifespan extension under caloric restriction in female mice^5^. Unfortunately, as males have not been used in that study, it is not clear if these conclusions are unique to females.

Altogether, these findings lend support to the idea that when caloric restriction and its mimetics are able to extend female lifespan, they do so not due to decrease in weight but by some other mechanism^17^; however, they almost always work in males, most likely via weight loss and/or growth retardation. This likely explains why most compounds that decrease weight are only able to increase lifespan in males but not in females. Given that female mice are lighter and live longer than males, one possibility is that life extension effects in males are, to some degree, due to changing their physiology to resemble females.

The significant sex-specific differences in median lifespan and weight change observed in our study have important implications for future research. It is crucial to include both male and female subjects in longevity studies to fully understand the effects of various compounds. Future research should aim to elucidate the underlying biological mechanisms that contribute to these differences, such as hormonal influences, metabolic rates, and genetic factors. Additionally, the potential confounding effects of drug-induced changes in feeding behaviour must be carefully controlled and monitored. Compounds that alter the taste, smell, or appearance of food, or that affect appetite and feeding behaviour through neuroendocrine pathways, could inadvertently induce caloric restriction, complicating the interpretation of results. Therefore, controlling for weight change while testing the effects of various compounds on lifespan is essential.

The updates in Build 5 of the DrugAge database have significantly enhanced the quality and usability of lifespan data, particularly from murine studies. The standardization of drug dosages, detailed recording of treatment protocols, and inclusion of weight change data provide a more robust framework for analysing the effects of compounds on lifespan. The improvements in the user interface, including new data columns and refined graphing options, allow for more precise and comprehensive analyses, facilitating better understanding and comparison of drug effects across different studies. Furthermore, our findings underscore the importance of controlling for weight change, as it can influence the outcomes of lifespan studies, especially in male mice. While not all drug-induced body weight reductions lead to life extension in mice, most substantial drug-induced life extensions occur together with body weight reduction. Importantly, the sex-specific differences observed in our analysis highlight the necessity of separate evaluations for males and females to interpret the effects of longevity interventions. Overall, these updates make the DrugAge database a more powerful tool for researchers investigating the pharmacological modulation of lifespan and pave the way for more accurate and reproducible findings in the field of aging research.

## Methods

Data were extracted manually from the source articles, as described in “*Build 5 updates”* section. Graphs with the corresponding statistics were created using custom Python scripts, using functions from the *scipy.stats* library, such as *linregress* for linear regression and *ttest_rel* for paired t-test.

## Supplementary information

SUPPLEMENTARY FIGURES_new

Supplementary table.

## Data Availability

The dataset used to generate the figures in this article is available as a Supplementary Table. The DrugAge database is available online (https://genomics.senescence.info/drugs/).
