# Supplementary material for: Sex-specific insights into drug-induced lifespan extension and weight loss in mice
Source: NPJ Aging. 2025 May 19;11(1):37. doi: 10.1038/s41514-025-00229-w (PMC12089533; doi:10.1038/s41514-025-00229-w)
Supplement: Supplementary file 1 — SUPPLEMENTARY FIGURES_new [file 41514_2025_229_MOESM1_ESM.pdf]

SUPPLEMENTARY FIGURES for

Sex-specific insights into drug-induced lifespan extension and weight loss in mice

Aleksey V. Belikov[1], Angelo Talay[1] and João Pedro de Magalhães[1*]

[1] Genomics of Ageing and Rejuvenation Lab, Department of Inflammation and Ageing, School of Infection, Inflammation and Immunology, College of Medicine and Health, University of Birmingham, UK.

* Corresponding Author

Corresponding Authors Contact Details:
Name: João Pedro de Magalhães
Address: Genomics of Ageing and Rejuvenation Lab, Department of Inflammation and Ageing, School of Infection, Inflammation and Immunology, College of Medicine and Health, University of Birmingham, Birmingham, UK
E-mail: jp@senescence.info

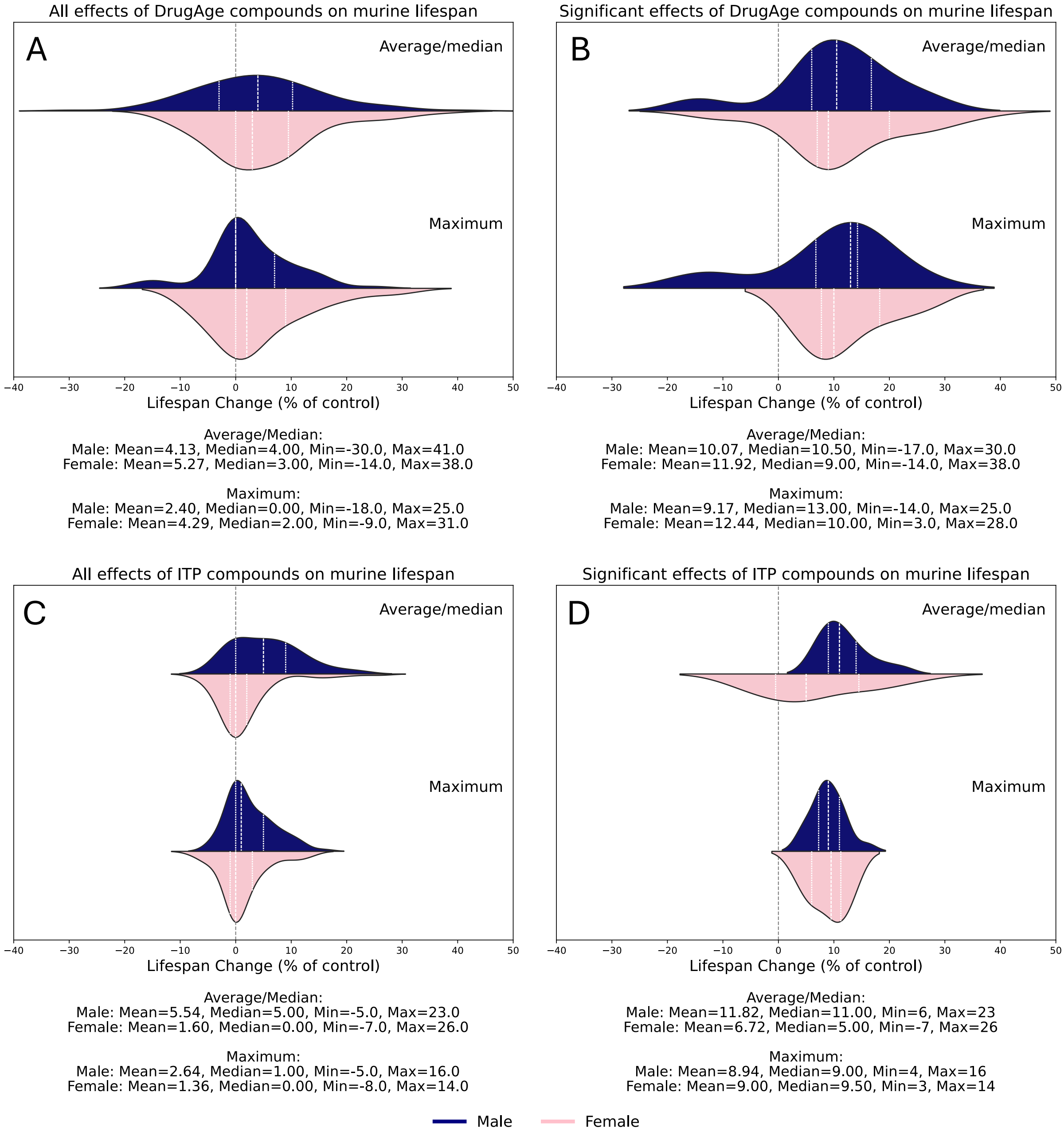

Supplementary Figure 1. Effects of compounds on average/median and maximum murine lifespan for males (top, navy) and females (bottom, pink). (**A**, **B**) Compounds from DrugAge. (**C**, **D**) Compounds from ITP studies. (**A**, **C**) All effects. (**B**, **D**) Significant effects. The significance of the effects was taken from the source publications.

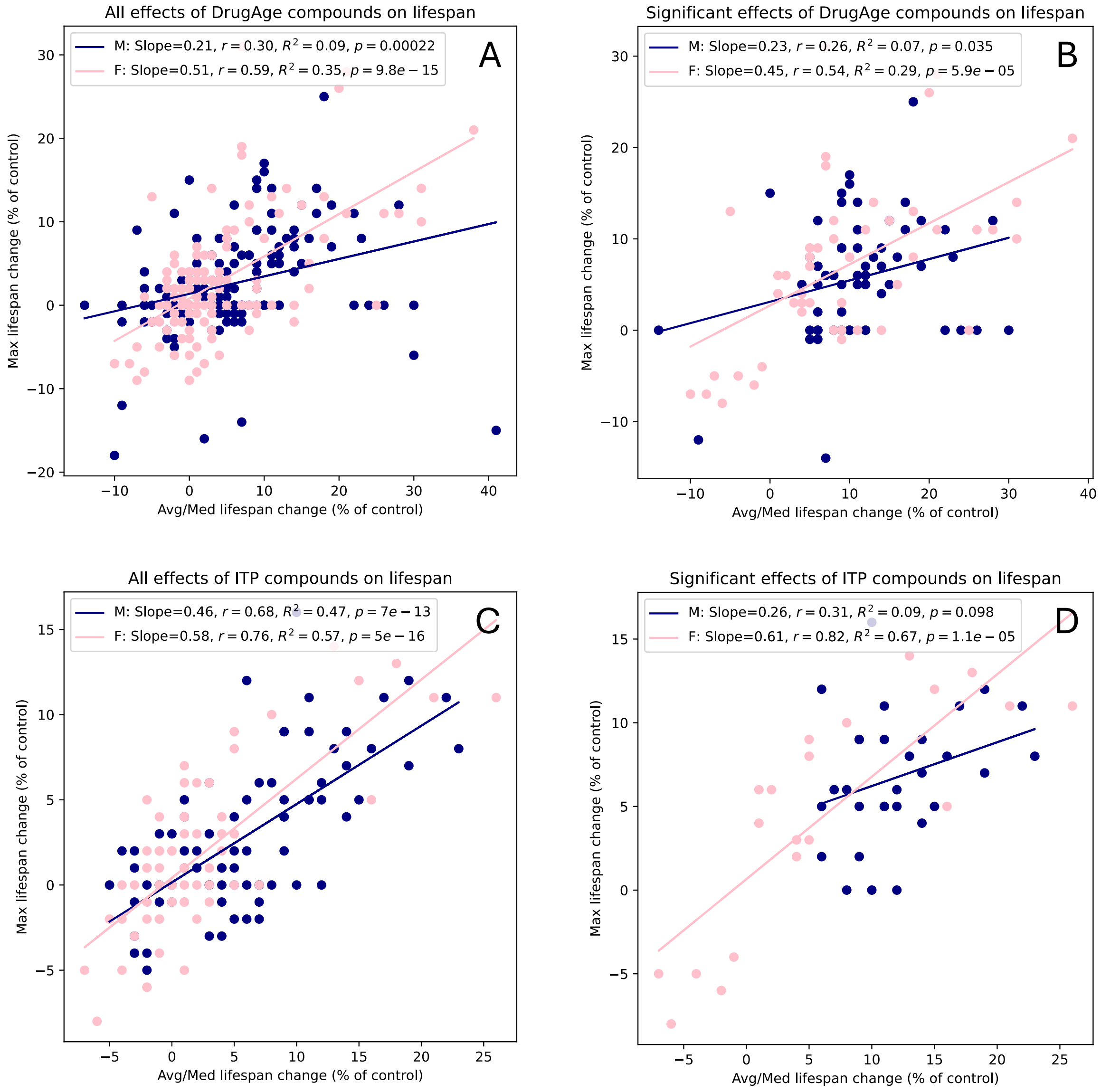

Supplementary Figure 2. Effects of compounds on average/median vs maximum murine lifespan for males (M, navy) and females (F, pink). (**A**,**B**) Compounds from DrugAge. (**C**,**D**) Compounds from ITP studies. (**A**,**C**) All effects. (**B**,**D**) Significant effects. The significance of the lifespan effects was taken from the source publications. The linear regression and the corresponding statistics were produced using the *linregress* function from the *scipy.stats* library in Python.

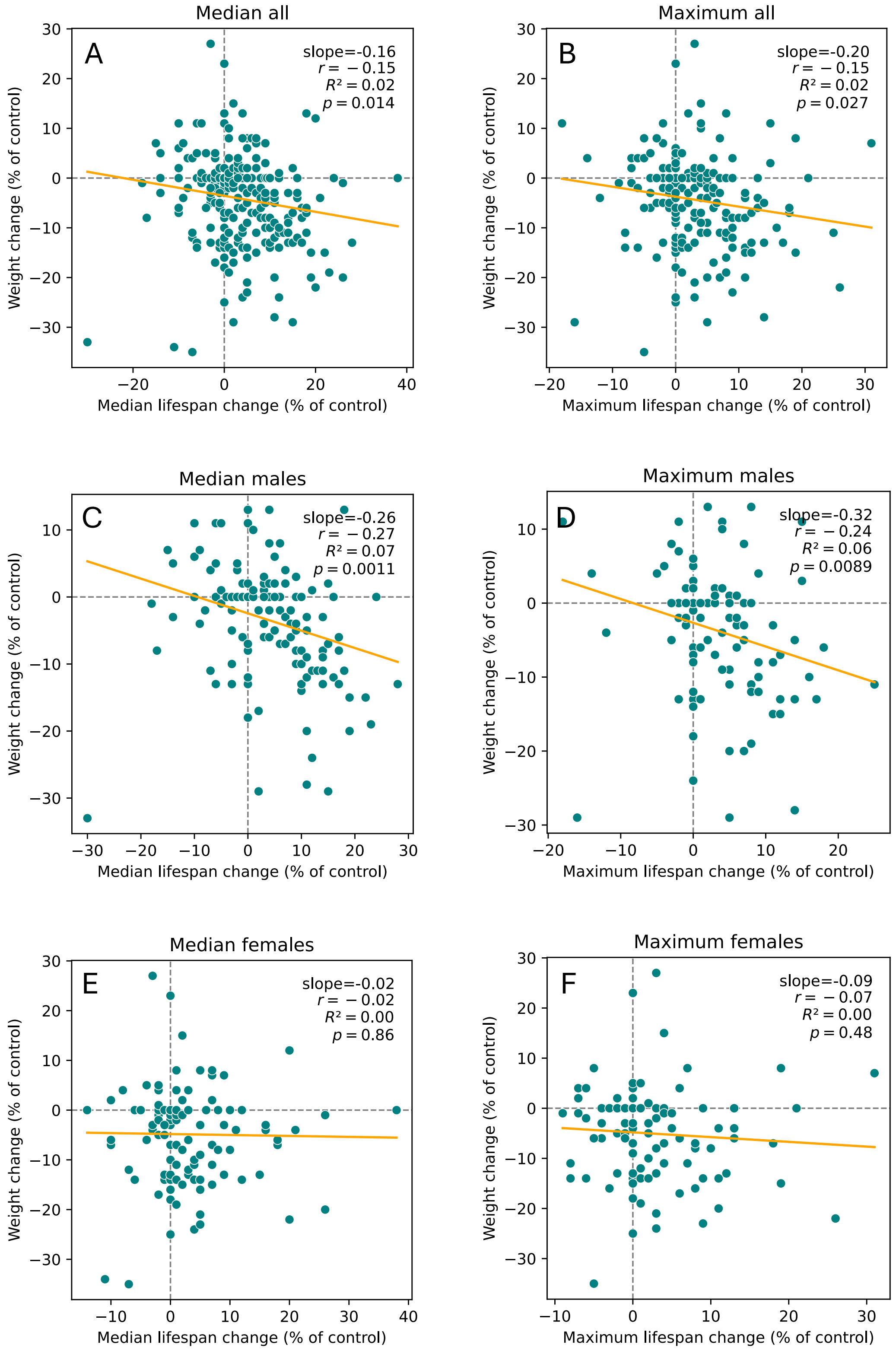

Supplementary Figure 3. Correlations between weight change and median (**A**,**C**,**E**) or maximum (**B**,**D**,**F**) lifespan change for males (**C**,**D**), females (**E**,**F**) and both sexes combined (**A**,**B**) from all available murine studies. The linear regression and the corresponding statistics were produced using the *linregress* function from the *scipy.stats* library in Python.

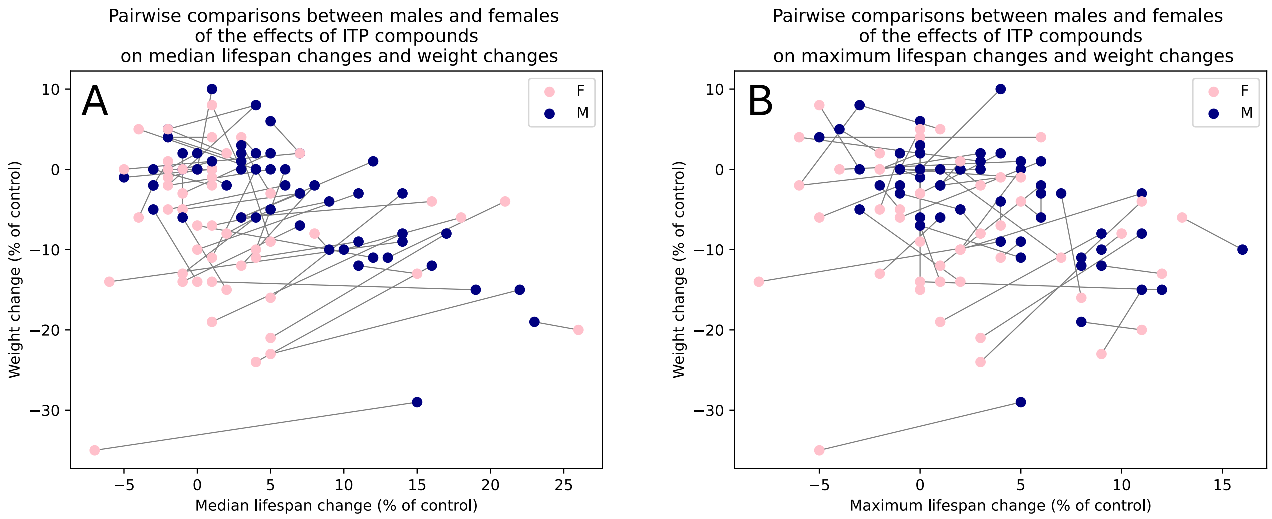

Supplementary Figure 4. Pairwise comparisons between males (M, navy) and females (F, pink) of the effects of compounds from ITP studies on median (**A**) or maximum (**B**) lifespan changes and weight changes. Lines represent individual compounds. All tested compounds were considered, including those with non-significant effects.
